# Social Support, Mindfulness, and Job Burnout of Social Workers in China

**DOI:** 10.3389/fpsyg.2022.775679

**Published:** 2022-02-17

**Authors:** Xiaoxia Xie, Yuqing Zhou, Jingbo Fang, Ganghui Ying

**Affiliations:** Research Institute of Social Development, Southwest University of Finance and Economics, Chengdu, China

**Keywords:** social workers, social support, mindfulness, job burnout, China

## Abstract

In the last 20 years, amid extensive social and economic reforms, China’s social structure and community life have changed considerably. A large number of social workers are needed to provide many more social services to community residents. The central government has issued many policies to rapidly develop human service organizations and increase the number of social workers. Thus, by the end of 2019, the number of social workers has reached more than 1.5 million in China. At the same time, local governments have issued many policies to promote an increase in the number of social workers. According to statistics from the Chengdu Civil Affairs Bureau, from 2010 to 2021, the number of social workers in Chengdu City increased, remarkably, from 553 to 17,622. Although the number of social workers has increased rapidly, some problems still exist. According to a survey by the Chengdu Social Workers Association, the turnover rate of social workers has reached approximately 20% in Chengdu City in 2018. Therefore, we aim to determine what influences social workers’ job burnout. Through regression analysis and mediation effect tests, we found the following: First, when controlling for gender, age, education, and workday, social support of social workers had a significant negative impact on job burnout (β = − 0.376). Second, the mindfulness of social workers had a significant negative impact on job burnout (β = − 0.320). Third, the mindfulness of social workers played a mediating role between social support and job burnout. The mediating effect was −0.116 (*p* < 0.001). Fourth, among the three dimensions of social support, mindfulness played a partially mediating role in family support and other support. The mediating effect between other support, which is the support from leaders and colleagues, and job burnout was the strongest, with a mediating effect of −0.109 (*p* < 0.001). In other words, the support provided by agency leaders and colleagues can maximize the level of mindfulness of social workers, thereby reducing social workers’ job burnout most effectively. We can thus reduce social workers’ job burnout by improving their level of mindfulness and the social support for them in China.

## Introduction

In recent decades, a lot of studies have discussed the stress, burnout, and turnover rate of helping professions (Lloyd et al., 2002; Pang et al., 2010; Yang et al., 2017; Jiang et al., 2019; Wang et al., 2021). Empirical research has shown that social workers may experience occupational stress, job burnout, and turnover intention (Soderfeldt et al., 1995; He, 2015; Lin and Deng, 2019; Zeng et al., 2019; Hu, 2021). However, despite the rapid development of the current social work industry, social workers in China still face a high turnover rate and job burnout (Jiang and Wang, 2016; Wang et al., 2019). Research has shown that job burnout is one of the strongest predictors of turnover intention (Mor Barak et al., 2001). Job burnout can cause anxiety and stress in social workers and gradually consume the individuals’ physical and mental resources. It is not conducive to the social workers’ physical and mental health (Durham, 1992; Shapiro et al., 2010). The job burnout of social workers has become an urgent problem to be solved for China. Despite this, little research examines the factors affecting job burnout of Chinese social workers (Huang et al., 2021). Therefore, this study focuses on support-related factors that contribute to and protect against job burnout in a sample of Chinese social workers from Chengdu, China. We also examine how mindfulness serves as a mediator in such relations. The results of this study not only can further enrich the research on factors affecting job burnout, but also can further alleviate job burnout among Chinese social workers and promote the sustainable and stable development of the Chinese social work industry.

## Literature Review

### The Current State of Social Work

In China, the economic reforms in the 1980s initially revealed a large number of social problems related to disadvantaged groups, which threatened the stability and cohesion of Chinese society (Ma, 2002). Therefore, amid the complexities and problems of economic transformation, social work has been restored and reconstructed in China (Liu, 2012). In 1988, the National Education Commission approved the opening of the Department of Social Work in the Department of Sociology of Peking University, focusing on the reconstruction of social work education. Since 1988, social work major in various universities has been a rapid development trend (Liu, 2001; Law and Gu, 2008). In 2015, There were a total of 1,842 MSW teachers nationwide, including 1,265 full-time teachers, and about 11–12 MSW professional teachers in various universities (Xie, 2017). As of the end of 2018, there were 348 undergraduate social work programs, 147 Master of Social Work programs, and 17 Ph.D. programs in China, with more than 40,000 graduates each year (Chan et al., 2020). At the end of 2018, there were 816,000 social organizations nationwide. Compared with 762,000 in 2017, the total number has increased by 54,000, the growth rate was 7.1%, and the growth rate has dropped by about 1.3% (Report on Social Organizations in China, 2019).

In Chengdu, Southwest Petroleum University took the lead in opening social work major. From 2002 to 2004, seven universities in the province successively opened social work major and recruited undergraduates majoring in social work. Since 2005, more than 10 universities in the province have established undergraduate and master’s programs in social work (Jiang, 2013). Following the 5.12 Wenchuan Big Earthquake in 2008 in Sichuan Province, social work experts and social workers from all over the country went to Sichuan to carry out social work services, which greatly promoted the development of social organizations in Chengdu and the growth of social workers (Huang, 2020). Meanwhile, the loss of social workers is also a common phenomenon. For example, the turnover rate of social workers in Shenzhen reached 22% in 2014 (Du, 2015), and the turnover rate of social workers in cities, such as Beijing, Shanghai, Nanjing (Li, 2016), and Guangzhou, also approached approximately 20% (Fang, 2015).

### Job Burnout

Maslach and Jackson (1981) defines job burnout as a symptom of encompasses exhaustion, cynicism, and sense of inefficacy in the occupational field, mainly occurs in human service professionals (Freuden-berger, 1974). Most domestic scholars use Maslach’s definition when studying job burnout and believe that such as heavy workload, lack of job recognition, and high work pressure are common causes of social workers suffering from job burnout (An, 2010; Sun and Liu, 2017; Tan, 2017). Burnout significantly positively predicts turnover rate (Mor Barak et al., 2001). Job burnout can cause anxiety and stress in social workers and gradually consume the individuals’ physical and mental resources, and it is not conducive to the social workers’ physical and mental health (Durham, 1992; Shapiro et al., 2010). It is very necessary to investigate the factors affecting burnout of Chinese social workers.

### Social Support and Job Burnout

Social support refers to the spiritual and material support given to individuals by organizations, family, relatives, friends, colleagues, partners, etc. It reflects the closeness and quality of a person’s social connections (Blumenthal et al., 1987), which have an impact on a person’s mental health. Social support can be divided into objective social support and perceived social support (Furmark et al., 2009). In this study, we combine Zimet et al. (1988) definition of perceived social support and defined perceived social support into the emotional experience that social workers experience through understanding and support from family, friends, and other sources. It is in a relatively stable state over a short period of time. A large number of studies have shown that social support is beneficial to individuals’ mental health (Cobb and Wills, 1985), and the discussion on the factors affecting social workers’ job burnout has mainly focused on the influence of different sources of social support. The theory of social support suggests that the survival of an individual depends on the support and help of others. The more social support an individual has, the better he or she can address the difficulties he or she faces. A large number of studies have also shown that there is a significant negative correlation between social support and job burnout. Related research has mainly discussed the impact of social support on job burnout according to the source and type of social support. For example, the study of Lee et al. (1990) confirmed that there was a negative correlation between social support and job burnout caused by work: among the various types, social support from leaders had the most obvious negative effect on job burnout. Schwarzer (1992) research on teachers found that compared with the support provided by family and friends, support from colleagues was more effective in reducing teachers’ job burnout. Sarros and Sarros (1992) mainly discussed the roles played by different types of social support. Their research implied that the actual support teachers received was more effective in reducing job burnout than emotional support. Feldman et al. (2002) believed that the higher the family support for employees in the transportation industry was, the less likely they were to suffer from job burnout.

Some research on the relationship between social support and job burnout has compared China to Western countries, but the research conclusions are basically consistent with the existing research conclusions in Western countries. Moreover, most Chinese scholars have found through their research that individuals’ social support was helpful to alleviate their job burnout. These studies have mainly concentrated on teachers, psychological counselors, medical staff, judges, and social workers because these groups have certain shared characteristics. For example, they have long and emotional working hours, highly emotional labor requirements, and a high susceptibility to external evaluation and self-evaluation (Yang et al., 2017); thus, they are more likely to develop job burnout. Lu (2015) stated in his research conclusion that the more social support elementary and middle school teachers received, the weaker their sense of job burnout. Qiao (2019) conducted questionnaire surveys and interviews with social workers in Chengdu and found that the higher the level of social support of social workers was, the weaker their feelings of job burnout. Zhang (2016) investigated a group of psychological counselors. He found that the social support obtained by this group can help alleviate burnout at work. The research content mainly focused on the relationship between social support, job burnout, and the influence mechanism between the two. Ma (2015) suggested that judges’ job burnout in China was generally more serious and that social support could produce more active coping styles, thereby reducing job burnout. Zhao et al. (2019) used a questionnaire survey method to show that social support could effectively reduce the job burnout of medical staff by enhancing the self-efficacy of medical staff in China.

### Social Support, Mindfulness, and Job Burnout

Previous studies of the mindfulness of social workers have focused more on the role of mindfulness in an intervention after job burnout occurs. Mindfulness in this article refers to a state of attention that is consciously aware of a moment without any judgment (Kabat-Zin, 2003). In this state, an individual will focus his or her consciousness and attention on current internal and external stimuli, including internal feelings and thoughts, as well as external sights and sounds, and accept these stimuli without evaluation (Baer et al., 2004). Different mindfulness states can be quantified by different mindfulness levels. Each person’s original mindfulness level is different, and each person’s mindfulness level is not fixed in different situations (Brown and Ryan, 2003).

In a study on sleep quality of college students, researchers found that students with high levels of perceived social support also had high levels of mindfulness, and mindfulness played a mediating effect between comprehension of perceived social support and sleep quality (Chen, 2018). In addition, perceived social support also has a positive impact on mindfulness. It means that firefighters with high levels of perceived social support have a high level of mindfulness. After facing the fire disaster and trauma, firefighters can show positive growth. Social support has a positive impact on mindfulness (Chen et al., 2020). Tan and Tao (2021) through a cluster sampling survey of doctors found that the perceived social support obtained by doctors had a positive impact on the improvement of their mindfulness level, and mindfulness played a part of the mediation between the perceived social support and job burnout. Through the positive emotional experiences of being supported, understood, and respected by individuals, social support provides encouragement and courage to overcome difficulties and challenges, thereby improving individuals’ self-satisfaction and confidence (Xie et al., 2019).

The mindfulness coping model entails that when an individual faces an external stimulus beyond his or her tolerance, the individual will adopt a decentralized adaptive response to his or her stress evaluation in a mindful way, focusing on the dynamic process of consciousness instead of the content of consciousness, thereby expanding his or her attention and strengthening his or her cognitive flexibility. Relying on their expanded metacognitive state, individuals actively re-evaluate stressful events, redefine or construct stressful events, and, finally, trigger positive feelings that can relieve stress, such as compassion, trust, self-confidence, or inner-peace (Garland et al., 2009). Continuous work pressure will quickly consume individuals’ physical and mental resources. When this unhealthy state persists, it will trigger emotional states, such as anxiety, depression, and burnout. However, if an individual can maintain abundant personal resources, such as mindfulness, he or she can effectively relieve negative emotions (Mesmer-Magnus et al., 2017). Recent studies have proven that mindfulness, as a personal psychological resource, plays a positive role in reducing people’s job burnout. Some studies showed that improving individuals’ mindfulness level can effectively alleviate the job burnout symptoms of social workers, such as insomnia, anxiety, and depression, thereby improving social workers’ physical and mental health (Brekke, 2012). Zeng and Qian (2017) found that the mindfulness of nurses was negatively associated with job burnout. Chen et al. (2018) used a five-factor mindfulness scale to test employees’ mindfulness level and concluded that employees’ mindfulness reduced job burnout. Cheng et al. (2020) found that the lower the level of mindfulness was, the more serious a teacher’s job burnout, which can further strengthen a teacher’s intention to resign.

**Figure 1:**
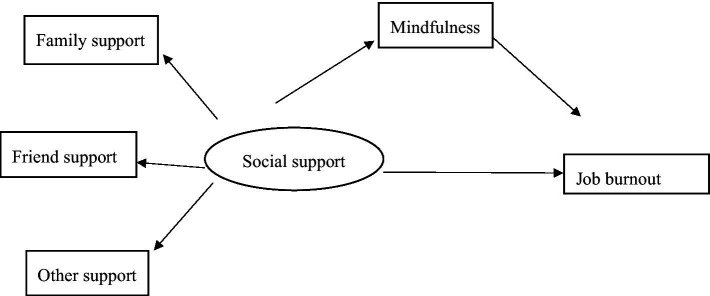
Relational model of social support, mindfulness, and job burnout.

In summary, mindfulness is affected by social support (Chen, 2018; Chen et al., 2020; Tan and Tao, 2021). In addition, mindfulness, as an important psychological resource, can reduce individuals’ job burnout, relieve bad emotions, and improve individuals’ physical and mental health (Brekke, 2012; Zeng and Qian, 2017; Chen et al., 2018, 2020). In the research on teachers, doctors, and other groups, mindfulness is generally used as a mediator (Chen, 2018; Tan and Tao, 2021). Social work is a typical helping profession, often accompanied by emotional labor, which can be seen as a high incidence of job burnout (Ren, 2017; Li, 2019). However, empirical research on the relationship between social support, mindfulness, and job burnout of Chinese social workers is still lacking. Thus, this article follows the perspective of social support theory to construct a model of the relationship between social workers’ social support, mindfulness, and job burnout (see Figure 1), proposing the following hypotheses:

*H1:* The social support of social workers has a negative correlation with job burnout.

*H2:* The level of mindfulness of social workers has a negative correlation with job burnout.

*H3:* The mindfulness of social workers plays a mediating role between social support and job burnout.

*H3-a:* The mindfulness of social workers plays a mediating role between family support and job burnout.

*H3-b:* The mindfulness of social workers plays a mediating role between friend support and job burnout.

*H3-c:* The mindfulness of social workers plays a mediating role between other support and job burnout.

## Materials and Methods

### Data and Sample

The data for the present study were collected from social workers in Chengdu, China, *via* an anonymous online survey. Chengdu is the city of Sichuan province and has a rapid development in social work. We randomly selected two districts out of 22 districts in Chengdu. With the help of Civil’s Affairs Bureaus (CAB), we successfully contacted social work professional associations and agencies to recruit participants within the two districts. Each district has around 600 social workers. On May 5, 2021, social workers of these associations and agencies were invited to participate in the survey. We sent reminders to participants 1 week and 2 weeks. On May 29, 2021, 915 social workers responded to the survey. We excluded 18 surveys from the final analysis due to incomplete and invalid data. The final sample contained from 897 social workers. The response rate of the survey was 75%. An informed consent process was implemented prior to the survey. Meanwhile, participants were informed that their participation was voluntary and that they could choose to stop the survey at any time. Every participant could compensate with 5RMB (1USD).

### Measures

The dependent variable, job burnout, was measured by the Maslach Burnout Inventory (MBI; Maslach et al., 1996). There are three versions of the MBI that are applicable to different groups of people: the MBI-Human Services Survey, MBI-Teacher Survey, and MBI-Universal Survey. Among them, the MBI-Human Services Survey (MBI-HSS) is suitable for people engaged in the service industry, including police, medical staff, and mental health workers. Social workers mainly supply persons with specialized social services, so the MBI-HSS is suitable for them. The original MBI-HSS had a total of 22 items, and after revision, 17 items were established. The survey contains three dimensions: emotional exhaustion (seven items; e.g., “I feel that my feelings have been exhausted at work”), depersonalization (three items; e.g., “Since taking this job, I have become more indifferent to people”), and personal accomplishment (seven items; e.g., “I can solve problems at work very effectively”). The scale uses a 1–7 grade scoring method, and the scores range from 1 (never) to 7 (every day). In three dimensions, high scores on the emotional exhaustion dimension and depersonalization dimension are indicative of burnout, and low scores on the personal accomplishment dimension are indicative of burnout. Thus, the personal accomplishment dimension items are reverse scoring (Florent et al., 2017). After reverse scoring, the higher the score of personal achievement dimension, the more serious the job burnout. The final total score of MBI includes the forward scoring of the first two dimensions and the reverse scoring of the third dimension. That is, high scores on the MBI are indicative of burnout. The scale total score ranges from 17 to 119 points. The Cronbach’s coefficient of the scale was 0.88.

The independent variable, perceived social support, was assessed by the perceived social support scale (PSSS; Jiang, 1999). It was based on the perceived social support scale compiled by Zimet et al. (1988). The scale mainly measures the social support perceived by individuals. It consists of three subscales and 12 self-assessment items. The scale consists of three subscales. Through factor analysis, each subscale has strong factorial validity, and subjects can clearly perceive and distinguish the three sources of social support (Zimet et al., 1988). The three subscales are family support (four items; e.g., “My family truly tries to help me”), friend support (four items; e.g., “My friends truly try to help me”), and other support (four items; e.g., “There is a special person who is around when I am in need”). The scale uses a 1–7 grade scoring method, and the scores range from 1 (strongly disagree) to 7 (strongly agree). The total score reflects the total degree of social support felt by the individual. The higher the total score is, the higher the individual’s level of social support. The scale total score ranges from 12 to 84 points. In our research, Cronbach’s alpha coefficients of the total scale and subscale were 0.95 for social support, 0.90 for family support, 0.93 for friend support, and 0.90 for other support.

We used Five Facet Mindfulness Questionnaire (FFMQ) to measure state mindfulness of samples (Baer et al., 2004). The original FFMQ has five dimensions and contains 39 items, which are evaluated from five dimensions: observation (eight items), description (eight items), conscious action (eight items), nonjudgment (eight items), and inaction (seven items). These items are based on a combination of exploratory factor analysis and confirmatory factor analysis of the MAAS, FMS, and KMS. Meng et al. (2020) based on an exploratory factor analysis and confirmatory factor analysis of the original FFMQ Scale, developed a Short-Form Five Facet Mindfulness Questionnaire (SF-FFMQ) specifically for China. The scale contains 20 items (e.g., “When I’m walking, I deliberately notice the sensations of my body moving”), which is also divided into five dimensions. Some items require reverse scoring (e.g., “I find myself doing things without intending to”). Participants are asked to score each item on the Likert scale, from 1 (not at all) to 5 (completely). The scale total score ranges from 20 to 100 points that reflects the individual’s mindfulness level. The higher the score is, the higher the individual’s mindfulness level. The Cronbach’s coefficient of the scale was 0.92.

### Analytical Strategy

We conducted analysis and Pearson’s correlation analysis, in order to observe the sample characteristics and the correlations among all variables. Then, we conducted regression analysis and bootstrap analysis to examine the relations among social support, family support, friend support, other support, mindfulness, and job burnout, while controlling gender, age, education, and working time. All control variables were assumed to have impact on mindfulness and job burnout. Bootstrap analysis repeats sampling of samples. Compared with the stepwise method of Baron and Kenny (1986), bootstrap analysis can obtain a more accurate confidence interval and a higher test power (Hayes and Scharkow, 2013; Kristopher et al., 2014). Therefore, this article combined the advantages of the stepwise method and bootstrap analysis, first through the regression analysis to test the coefficients, and then used the bootstrap analysis to re-test the indirect effects and confidence intervals to improve the test power and the explanatory power of the results. STATA15.0 software was used for all analyses.

## Results

Since the data in this study were derived from the subjects’ self-reports, there may be common method deviations. We used Harman’s single factor method for the common method deviation test. We found five factors with eigenvalues greater than one, and the variance explained by the first factor was 36.53%; less than the critical value of 40%. Our study had no serious common method deviation in the data.

Table 1 presents the descriptive statistics and correlations of key variables. The average age of the sample was 31.8 years old. About 78% were female. A large number of social workers had college and above degree (54.6%). The average weekly workdays of many social workers were approximately 5.2 days. The average score of social support obtained by the sample was 59.3. Among the participants, many felt the most support came from their friends (*M* = 20.1, SD = 4.4). Notably, the samples’ average mindfulness score was 61.9. The average score of job burnout of the sample was 53.9 (SD = 16.6).

**Table 1:** Descriptive statistics and correlations of key variables.

Variable	Mean (S.D.)	1	2	3	4	5	6	7	8	9	10
1. Female (0–1)	0.8 (0.4)	—									
2. Age (20–50)	31.8 (7.3)	0.04	—								
3. Education-College and above (0–1)	0.6 (0.5)	0.05	−0.32^***^	—							
4. Weekly workdays (0–7)	5.2 (0.5)	0.09^*^	0.11^**^	0.01	—						
5. Social support	59.3 (12.4)	−0.02	0.12^***^	−0.06	−0.002	—					
6. Family support	19.5 (4.9)	−0.04	0.17^***^	−0.08^*^	−0.03	0.89^***^	—				
7. Friend support	20.1 (4.4)	−0.03	0.06	−0.02	0.002	0.92^***^	0.72^***^	—			
8. Other support	19.6 (4.4)	0.01	0.09^**^	−0.06	0.02	0.90^***^	0.68^***^	0.79^***^	—		
9. Mindfulness	61.9 (6.5)	0.03	0.11^***^	0.09^**^	0.06	0.37^***^	0.32^***^	0.35^***^	0.33^***^	—	
10. Job burnout	53.9 (16.6)	0.09^*^	−0.16^***^	0.13^***^	0.07^*^	−0.40^***^	−0.36^***^	−0.34^***^	−0.37^***^	−0.41^***^	—

N = 897, ^*^p < 0.05, ^**^p < 0.01, and ^***^p < 0.001.

The correlations of the key variable results are shown in Table 1. Age was positively correlated with social support (r = 0.12, *p* < 0.001), family support (r = 0.17, *p* < 0.001), and other support (r = 0.09, *p* < 0.01). Education was negatively correlated with family support (r= − 0.08, *p* < 0.05). In addition, age (r = 0.11, *p* < 0.001), education (r = 0.09, *p* < 0.01), social support (r = 0.37, *p* < 0.001), family support (r = 0.32, *p* < 0.001), friend support (r = 0.35, *p* < 0.001), and other support (r = 0.33, *p* < 0.001) were all positively correlated with mindfulness. Ultimately, regarding job burnout, the older the social worker was, the lower the level of job burnout (r = − 0.16, *p* < 0.001). The higher the educational background was, the more serious the job burnout (r = 0.13, *p* < 0.001), and the longer the weekly workdays was, the more serious the job burnout (r = 0.07, *p* < 0.05). In general, social support (r = − 0.40, *p* < 0.001), family support (r = − 0.36, *p* < 0.001), friend support (r = − 0.34, *p* < 0.001), and other support (r = − 0.37, *p* < 0.001) were all negatively correlated with job burnout. The higher the level of mindfulness of social workers was, the more likely they were to reduce their own burnout (r = − 0.41, *p* < 0.001).

Table 2 presents the regression analysis of the 3 models. Model 1 analyzed the impact of social support on job burnout. The results showed that adj.*R*^2^ = 0.18, *F* = 40.84, *p* = 0.000; social support was negatively associated with job burnout (β = − 0.376, *p* < 0.001). That is, the higher the level of social support that social workers received, the lower their level of job burnout. Model 2 analyzed the impact of social support and mindfulness on job burnout, adj.*R*^2^ = 0.27, *F* = 55.40, *p* = 0.000, indicating that the goodness of fit of Model 2 is higher than that of Model 1. Social support was negatively associated with job burnout (β = − 0.260, *p* < 0.001), and mindfulness was also negatively associated with job burnout (β = − 0.320, *p* < 0.001). Thus, the results of Model 1 and Model 2 verified Hypothesis 1 and Hypothesis 2. Furthermore, Model 3 analyzed the three dimensions of social support, including family support, friend support, and other support’s effects on job burnout. The results showed that family support (β = − 0.124, *p* < 0.01), other support (β = − 0.183, *p* < 0.001), and mindfulness (β = − 0.321, *p* < 0.001) were negatively associated with job burnout.

**Table 2:** Regression analysis of job burnout.

Variable	Model 1			Model 2			Model 3		
	β	SE	*P*	β	SE	*P*	β	SE	*P*
Social support	−0.376	0.030	^***^	−0.260	0.031	^***^			
Family support							−0.124	0.043	^**^
Friend support							0.017	0.051	
Other support							−0.183	0.048	^***^
Mindfulness				−0.320	0.031	^***^	−0.321	0.031	^***^
Gender	0.071	0.030	^*^	0.077	0.029	^**^	0.080	0.029	^**^
Age	−0.106	0.032	^**^	−0.070	0.031	^*^	−0.067	0.031	^*^
Education	0.069	0.032	^*^	0.116	0.031	^***^	0.113	0.031	^***^
Workday	0.078	0.031	^*^	0.093	0.029	^**^	0.093	0.029	^**^
*N*	897			897			897		
*F*	40.84^***^			55.40^***^			42.37^***^		
Adjusted *R*^2^	0.18			0.27			0.27		

N = 897, ^*^p < 0.05, ^**^p < 0.01, and ^***^p < 0.001.

Furthermore, regression analysis indicated that among the control variables, gender, age, education, and workday all passed the significance test in the three models, indicating that the four variables all have a significant association with job burnout. For example, in Model 1, gender (β = 0.071, *p* < 0.05), education (β = 0.069, *p* < 0.05), and workday (β = 0.078, *p* < 0.05) were positively associated with job burnout. In contrast, age was negatively associated with job burnout (β = − 0.106, *p* < 0.01). Thus, according to the regression coefficients of the three support dimensions, other support had the greatest impact on social workers’ job burnout, followed by family support, but friend support had no significant correlation with job burnout.

### Mediating Role of Mindfulness

**Figure 2:**
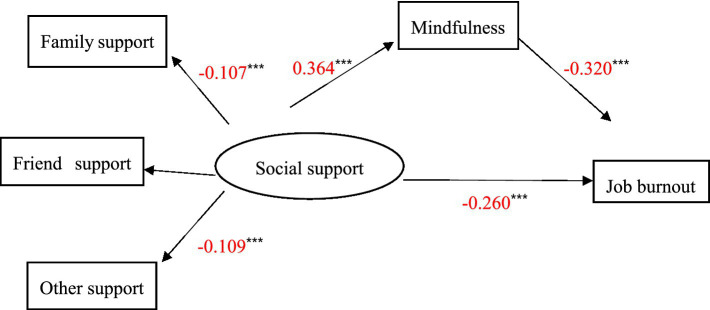
Standardized estimates of social support, mindfulness, and job burnout. The *** means *p* < 0.001.

Under the condition of controlling for gender, age, education, and workday, we tested whether mindfulness can mediate the relationship between social support and job burnout. Table 3 and Figure 2 suggested that after adding the intermediary variable mindfulness, social support had a significant negative predictive effect on job burnout (β = − 0.260, *p* < 0.001), and the coefficient was less than the original −0.376. The total effect of social support on job burnout was −0.376, and the indirect effect of social support *via* mindfulness was −0.116 (*p* < 0.001). Thus, mindfulness played a partial mediating role in the impact of social support on job burnout. The mediating effect was −0.116, the 95% bootstrap confidence interval was [−0.148, −0.088], and the proportion of the effect mediated by mindfulness was 0.309 (−0.116/−0.376). This verified Hypothesis 3. We further tested the mediating role of mindfulness in the impact of different dimensions of social support on job burnout; Table 3 illustrated that mindfulness played a partial mediating role in the impact of family support on job burnout. The mediating effect was −0.107 (*p* < 0.001), the 95% bootstrap confidence interval was [−0.139, −0.082], and the proportion of family support’s effect on job burnout that was mediated by mindfulness was 0.318 (−0.107/−0.337). In addition, mindfulness played a similarly partial mediating role in the impact of other support on job burnout. The mediating effect was −0.109, the 95% bootstrap confidence interval was [−0.142, −0.082], and the proportion of other support’s effect on job burnout that was mediated by mindfulness was 0.304 (−0.109/−0.359). Accordingly, Hypotheses H3-a and H3-c were verified.

**Table 3:** Bootstrap analysis of mediation effect test.

Path	Estimate	SE	Boot CI (95%)
Social support→Mindfulness→Job burnout	−0.116^***^	0.015	[−0.148, −0.088]
Family support→Mindfulness→Job burnout	−0.107^***^	0.015	[−0.139, −0.082]
Other support→Mindfulness→Job burnout	−0.109^***^	0.015	[−0.142, −0.082]

N = 897, ^*^p < 0.05, ^**^p < 0.01, and ^***^p < 0.001.

In summary, mindfulness played a mediating role between the social support of social workers and job burnout. Through further analysis, it was found that mindfulness also played a partially mediating role between the two dimensions of family support and other support of social workers and job burnout. Moreover, mindfulness had the strongest mediating effect between other support and job burnout. Therefore, the support provided by agency leaders and colleagues can maximize individuals’ level of mindfulness, thereby more effectively reducing social workers’ job burnout.

## Discussion

Empirical research from studies mainly focused on samples of teachers, doctors, etc. It has shown that social support is beneficial to individuals’ mental health (Cobb and Wills, 1985) and reduce job burnout (Halbesleben, 2006; Salami, 2009; Zhu et al., 2013). Research also showed that mindfulness, as a personal psychological resource, plays a positive role in reducing individuals’ job burnout (Brekke, 2012; Zeng and Qian, 2017; Chen et al., 2018). Less is known about whether the mediation effect of mindfulness in the relation between social support and job burnout is in social workers. This study examined how social support and different dimensions were related to job burnout and whether these relations were mediated by mindfulness in social workers. Therefore, the present study extends the literature by investigating the mediation effect of mindfulness in the relation between social support and job burnout in social workers.

The descriptive statistics indicated that social workers had long working hours, heavy workloads, and high work intensity on the job. The regression analysis results provided support for the hypothesized process of job burnout in social workers. The results showed that the high level of social support received by social workers was correlated with a low level of job burnout (β = − 0.376), which was consistent with previous research results (Qiao, 2019). The improvement in the level of social support promotes individuals to overcome difficulties from their external environment and enhances their motivation to cope with stressful events (Shi and Shi, 2013). Moreover, we also found that among the three dimensions of social support, family support (β = − 0.124), and other support (β = − 0.183) were significantly negatively associated with job burnout. Of them, the support provided by leaders and colleagues was the most helpful in reducing job burnout, which was consistent with the conclusions of existing research (Schwarzer, 1992; Qu et al., 2013; Zhang et al., 2014). In addition to serviced persons, social workers spend the most time with their colleagues and leaders in their daily work. When they face great pressures and difficulties at work, their family and friends cannot help them solve practical work problems in addition to providing them with spiritual support. However, leaders and colleagues can provide more actual support, which includes sharing a workload and providing practical solutions at work (Liu et al., 2018). Leaders and colleagues can help social workers solve some actual difficulties during workdays. When most of their problems at work can be resolved under the support provided by leaders and colleagues, social workers will experience less work pressure and feel better. In this way, the job burnout of social workers can be relieved to a large extent (Feng and Wang, 2014; Kong, 2017).

Importantly, mindfulness was found to act as a mediator of relation between social support and job burnout, pointing to mindfulness as a key point of intervention, which was consistent with the existing research results (Tan and Tao, 2021). On the one hand, the results showed that social support was positively correlated with mindfulness (*β* = 0.364), which was consistent with previous research results (Huang et al., 2021). In their research, support from colleagues of social workers can be seen as a job resource, which enables social workers to have a better state of mindfulness. Tan and Tao (2021) through a cluster sampling survey of doctors found that the perceived social support obtained by doctors had a positive impact on the improvement of their mindfulness level. In a study of the relationship between social support and better mental health, there is a positive correlation between social support and mindfulness (Klainin-Yobas et al., 2016; Mettler et al., 2019). Ali et al. (2019) showed that students who had better social support had more mindfulness. Besides, a study also found that social support is the main influencing factor of the level of mindfulness of pregnant women with threatened preterm labor. Good social support can provide a buffer and protect, promote physical and psychological adaptation of the individual, and promote positive behavior change, so as to conduct more effective self-behavior regulation, thereby improving mindfulness level. The higher the level of social support, the higher the level of mindfulness (He et al., 2021). On the other hand, when individuals have a high level of mindfulness, it can effectively reduce the job burnout that occurs at work (Bonifas and Napoli, 2014; β = − 0.320). In this study, mindfulness of social workers was significantly negatively correlated with job burnout, which was consistent with the findings of Brekke (2012). Mindfulness can help social workers receive enough emotional energy and create emotional space, allowing them to accept their shortcomings and limitations, and let go of their prejudices against external criticism or self-judgment (Birnbaum and Birnbaum, 2008). At the same time, the improvement of the level of mindfulness can reduce the emotional fatigue and occupational stress of social workers, thereby improving psychological flexibility (Gail et al., 2012). In addition, the mediating effect results further showed that mindfulness played a partial mediating role between social workers’ family support, other support, and job burnout. Of these, mindfulness had the greatest mediating effect on the impact of other support on job burnout (indirect effect was −0.109). When social workers are faced with work challenges, their leaders and colleagues of human service organizations provide them with substantial assistance to the greatest possible extent on the work, which can help them to positively release occupational stress and generate positive emotions, and, ultimately, reduce job burnout (Tang and Ye, 2007; Zhang and Liu, 2010; Zhao, 2016).

Based on our all findings of our study, the level of mindfulness of social workers can be improved, social workers’ job burnout can be alleviated efficiently. We should give attention to not only the role of social workers’ social support in relieving job burnout but also to improving the level of mindfulness of social workers. We offer a few practice suggestions for social workers, human service organizations, and the government. These three should make collective efforts to reduce job burnout. First, social workers should further strengthen their personal qualities and abilities and rely on their own efforts to obtain the support of their families, leaders, and colleagues. Second, human service organizations should adopt some methods to reduce the work pressure on social workers, such as cultivating social workers’ mindfulness, organizing team building activities, raising wages, and taking care of their mental health. Third, local governments should issue some policies to strengthen the publicity of social workers, which can improve the professional identity of social workers.

This article follows the perspective of social support theory to construct a model of the relationship between social workers’ social support, mindfulness, and job burnout. To summarize, it does contribute to further understanding the social workers’ job burnout and extends the literature by investigating the mediation effect of mindfulness in the relationship between social support and job burnout in developing countries. Moreover, our findings provide empirical evidence and useful opinions for alleviating job burnout of social workers, enhancing social support for social workers, and promoting the sustainable and stable development of the social work industry in reality.

The results of this study have some limitations. First, the data mainly come from social workers’ subjective reports, which may include some subjects’ memory bias. Second, the data all belongs to cross-sectional data. If we want to know the long-term effects of social support and mindfulness on job burnout, we need to track the sampled individuals and collect long-term data. Fortunately, this paper is a useful beginning that allows us to analyze the negative influence of social support and mindfulness on job burnout. If we want to reduce the job burnout of social workers, we should improve their social support, especially leaders’ and colleagues’ support, and adopt some methods to improve the mindfulness of social workers. Future research may consider collecting data from multiple sources, such as social workers’ families, friends, leaders, and colleagues, to compare subjective and objective data. Longitudinal research could also be considered to test the stability of the relationship among social support and mindfulness and job burnout.

## Conclusion

Based on intermediary model, this study analyzed the relations among social support and dimensions, job burnout in a sample of 897 social workers from China. Furthermore, we explored whether mindfulness mediates the relations between social support and dimensions, and job burnout. The results demonstrate how social support affects social workers’ job burnout. Our empirical findings show that social support has a significantly negative association with job burnout. On the one hand, social support has a significant negative impact on social workers’ job burnout. On the other hand, social support can also indirectly predict job burnout through mindfulness. Thus, mindfulness intervention may be useful for social workers to reduce or avoid their job burnout.

## Data Availability Statement

The original contributions presented in the study are included in the article/supplementary material, further inquiries can be directed to the corresponding author.

## Ethics Statement

Written informed consent was obtained from the individual(s) for the publication of any potentially identifiable images or data included in this article.

## Author Contributions

XX, YZ, JF, and GY: conceptualization and writing—review and editing. XX and YZ: methodology, software, and validation. YZ: analysis and data curation. YZ, JF, and GY: investigation. YZ and JF: writing—original draft preparation. XX: resources. All authors contributed to the article and approved the submitted version.

## Conflict of Interest

The authors declare that the research was conducted in the absence of any commercial or financial relationships that could be construed as a potential conflict of interest.

## Publisher’s Note

All claims expressed in this article are solely those of the authors and do not necessarily represent those of their affiliated organizations, or those of the publisher, the editors and the reviewers. Any product that may be evaluated in this article, or claim that may be made by its manufacturer, is not guaranteed or endorsed by the publisher.
